# Secondary analysis of California regional water boards dataset: Morphological distribution of aquatic microplastics in San Francisco Bay and implications for liver disease pathogenesis

**DOI:** 10.1016/j.iliver.2026.100254

**Published:** 2026-07-13

**Authors:** Muhammad Adil Malik, Quan Zhuang

**Affiliations:** a Transplantation Center, The Third Xiangya Hospital of Central South University, Changsha, 410013, China; b Key Laboratory of Translational Research in Transplantation Medicine of National Health, Commission, The Third Xiangya Hospital of Central South University, Changsha, 410013, China

**Keywords:** Microplastic, Morphology, Data analysis, Public data, Liver disease, Pathogenesis, Fiber, Fragment

## Abstract

**Background and aims:**

Microplastics (MPs) are widespread environmental pollutants, and there is growing evidence that they have potential impacts on human health. However, the role of particle morphology in mediating biological responses, particularly in liver disease, is still poorly understood. The aim of this study was to quantify the morphological distribution of MPs in aquatic environments and to assess their potential biological relevance based on experimental evidence.

**Methods:**

A secondary analysis was conducted using a publicly available dataset of 19,874 MPs collected from surface waters of San Francisco Bay (2015–2020). Morphological, size, color, and spatial data were analyzed using R (v4.3.1). Temporal trends, spatial autocorrelation, and size-geometry relationships were assessed.

**Results:**

Fibers (49.8%) and fragments (40.3%) dominated the dataset. A significant temporal increase in fiber abundance was observed between 2015 and 2018 (*p* = 0.012). These fibers had extreme aspect ratios (> 50 : 1), and spatial clustering was identified in the Central Bay (*p* < 0.001).

**Conclusion:**

These results suggest that microplastic morphology, particularly high-aspect-ratio fibers and irregular fragments, is a critical parameter in assessing environmental exposure and provides biological plausibility for oxidative stress and inflammatory pathways relevant to liver disease pathogenesis.

## Abbreviations

CLSM: Confocal Laser Scanning Microscopy
CYP450: cytochrome P450
FTIR: Fourier Transform Infrared Spectroscopy
IL-6: interleukin-6
LPS: lipopolysaccharide;
MAFLD: metabolic dysfunction-associated fatty liver disease
MET: macrophage extracellular trap;
MP: microplastic
NAFLD: non-alcoholic fatty liver disease
PPAR: peroxisome proliferator-activated receptor
qPCR: quantitative polymerase chain reaction
ROS: reactive oxygen species
scRNA-seq: single-cell RNA sequencing
SEM: scanning electron microscopy
TEM: transmission electron microscopy
TNF-α, tumor necrosis factor alpha

## 1 Introduction

Microplastics (MPs), plastic particles measuring less than 5 mm, have emerged as a widespread environmental pollutant and are of global concern.^1^ With annual plastic production exceeding 300 million tons, a significant portion of plastic waste enters natural ecosystems, where it fragments into persistent MPs.^2^ These particles are highly resistant to degradation and are now widespread in aquatic, terrestrial, and atmospheric environments, raising growing concerns about their long-term ecological and health impacts. Human exposure to microplastics is considered unavoidable. MPs have been detected through various exposure routes, including food, drinking water, and air, and have been identified in human biological samples such as blood, bone and placental tissue.^3^^,^^4^ This widespread presence indicates the potential for chronic exposure at low levels in various populations.^5^ However, despite growing evidence of environmental prevalence, the implications of such exposure for human health remain unclear.

The liver, as a central organ involved in metabolism, detoxification, and immune regulation, represents a plausible target for microplastic-related effects.^6^ These findings suggest a potential relevance to liver pathological processes, although direct evidence in humans remains limited.^7^ Experimental studies in animal models have shown that microplastics can cross the intestinal barrier, enter the systemic circulation, and accumulate in liver tissue.^8^^,^^9^ Such exposures have been associated with oxidative stress, inflammatory responses, and metabolic disorders, including lipid dysregulation and steatosis.^10^^,^^11^ It is important to emphasize that microplastics are not a uniform class of pollutants, and that their biological effects depend more on their physical and chemical characteristics. Among these characteristics, particle morphology (shape) has emerged as a crucial, but still understudied, factor in cellular interactions.^12^ Experimental studies have shown that elongated particles with a high aspect ratio, such as fibers, can cause incomplete phagocytosis and a prolonged inflammatory response, and that irregular fragments can induce various mechanical and cellular stress effects.^13^ Despite this evidence, there is still a discrepancy between detailed environmental characterization of microplastic morphology and the specific shape-related mechanisms reported in toxicological studies.

Large-scale analyses measuring the morphological distribution of microplastics in environments relevant to exposure are still limited. It is unclear whether the most abundant environmental particle shapes correspond to those shown to induce significant biological responses in experimental systems.^14^^,^^15^ Filling this gap is crucial to improving the relevance of environmental data for human health risk assessment.

Therefore, this study aims to analyze a large, publicly available dataset of microplastics present in surface waters of San Francisco Bay to characterize their morphological distribution, temporal trends, spatial patterns, and size-geometry relationships. The results are interpreted in the context of experimentally reported shape-dependent biological mechanisms to explore their potential relevance to liver disease pathogenesis. This study integrates environmental morphological data with experimentally reported shape-dependent biological mechanisms to provide a framework for assessing potential health relevance.

## 2 Materials and methods

### 2.1 Sample collection

The dataset was obtained from the California Water Boards Microplastic Data repository (https://catalog.data.gov/dataset/microplastic-and-microparticle-data-from-surface-water-san-francisco-bay-and-adjacent-sanc). This repository was selected because it provides a large (*n* = 19,874), publicly available, morphology-annotated microplastic dataset with standardized collection (manta trawl, 333 μm mesh) and polymer verification (FTIR spectroscopy), and because San Francisco Bay represents a well-characterized urban estuary relevant to human exposure pathways. The data span 2015–2020. Extracted variables included morphology, color, size, and polymer type. Records with missing morphological data were excluded, and the mislabeled category “Fear” was corrected to “Foam” following terminology validation. Sampling locations were georeferenced using high-precision GPS to support spatial analysis. Quality control included procedural blanks to monitor airborne contamination, pre-rinsing of glassware with filtered water, and polymer verification via Fourier-transform infrared spectroscopy.

### 2.2 Environmental parameters

During the collection phase, key environmental parameters, including water pH and turbidity, were measured using calibrated multiparameter probes. Analysis of these parameters provided insight into their potential influence on MP dynamics and distribution patterns.

### 2.3 Quality control and contamination prevention

The study implemented measures to maintain the integrity and reliability of the data collected regarding MPs in aquatic environments. To prevent contamination during sample collection and analysis, all glassware and instruments used in the study were pre-rinsed with filtered water prior to use. This step helps minimize the risk of introducing external contaminants that could affect the results. In addition, procedural blanks were included in each batch of samples to monitor and quantify potential contamination from the environment. These blanks served as controls to assess airborne contamination and any inadvertent introduction of non-target materials during the sampling and analytical processes.

### 2.4 Ethical considerations

This study was based exclusively on a publicly available environmental microplastic dataset from the California Water Boards Microplastic Data repository via Data.gov. No human participants, human biological materials, animals, or identifiable personal information were involved. Therefore, institutional review board approval and informed consent were not required.

### 2.5 Statistical analysis and environmental correlations

Data were analyzed using R software (version 4.3.1; R Foundation for Statistical Computing, Vienna, Austria), available at https://www.r-project.org/. Descriptive statistics summarized MP concentrations and morphological distributions. Temporal trends in morphological composition were examined using linear regression. Relationships between morphological categories and color were assessed using a chi-square test of independence. Spatial autocorrelation of MP concentrations was assessed using Moran's I statistic. Differences in particle size distributions between morphological categories were tested using the nonparametric Kruskal-Wallis test, followed by post-hoc pairwise comparisons where appropriate. Relationships between MP abundance and environmental parameters (pH and water turbidity) were examined using Pearson's correlation coefficient (*r*). All statistical tests were two-tailed, with a significance level set at *p* < 0.05.

## 3 Results

### 3.1 Morphological distribution and environmental characteristics

**Fig. 1:**
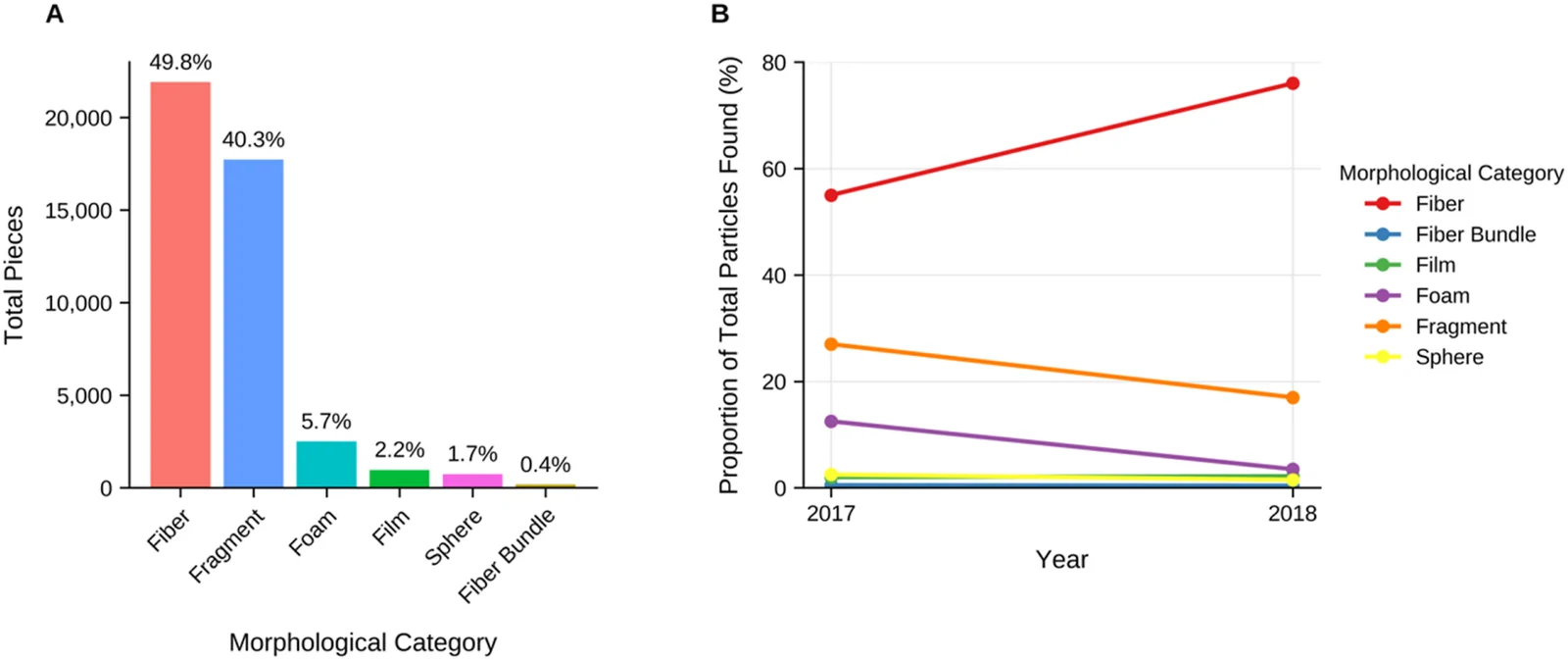
Morphological distribution and temporal trends of microplastics in San Francisco Bay surface waters (2015–2018). (A) Overall distribution (*n *= 19,874 particles): irregular fibers (49.8%) and fragments (40.3%) predominated and together accounted for over 90% of all particles. (B) Temporal trend: fiber abundance increased significantly from 55% to 78% (linear regression slope = 5.8% per year, *p *= 0.012).

Analysis of 19,874 individual microplastic particles revealed a highly skewed morphological distribution, with fibers (49.8%) and fragments (40.3%) predominating, which together constituted over 90% of all observed particles (Fig. 1A). Minor categories included foam (5.7%), films (2.2%), spheres (1.7%), and fiber bundles (0.4%).

**Fig. 2:**
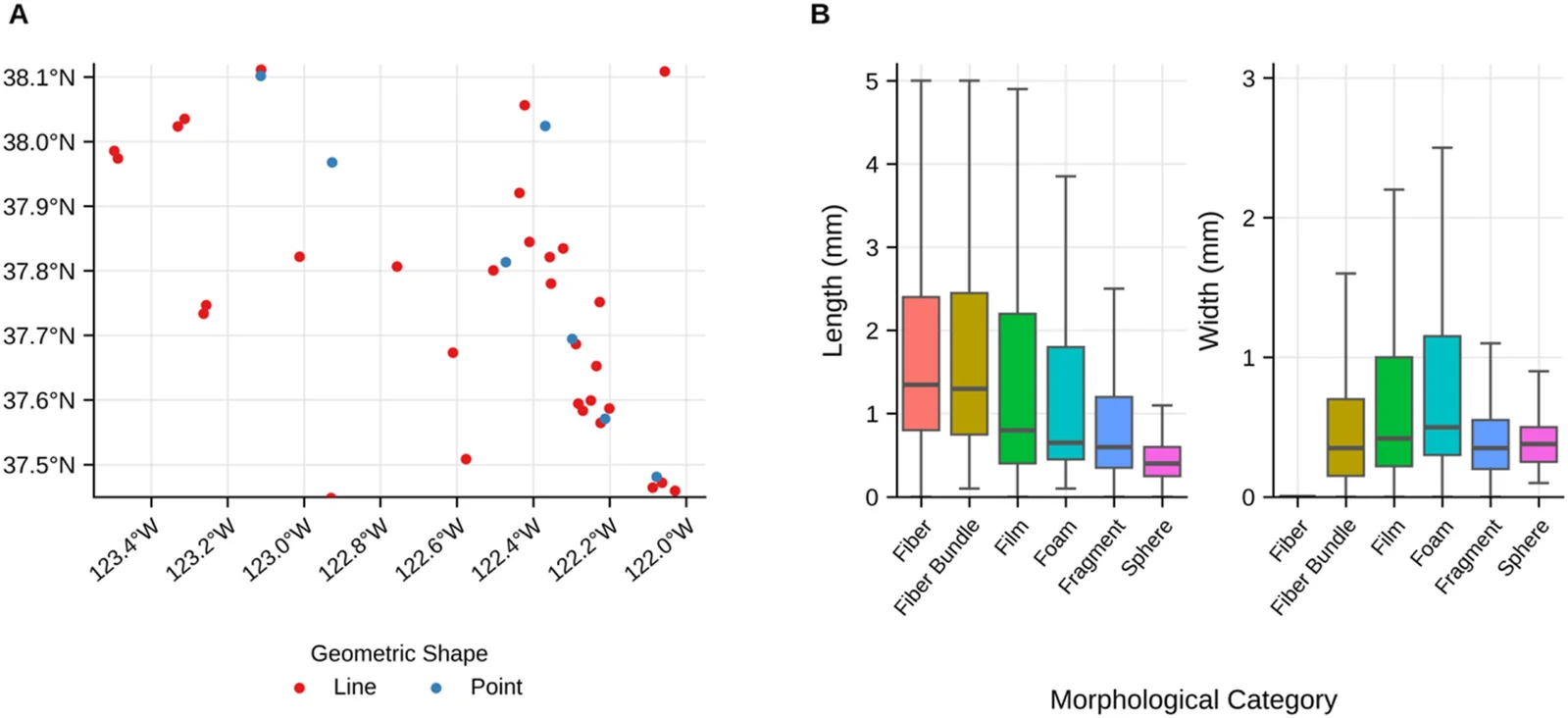
Spatial distribution and size-geometry characteristics of microplastics in San Francisco Bay. (A) Spatial clustering of sampling sites. Significant clustering occurred in the Central Bay (Moran's I = 0.32, *p* < 0.001), with mean concentrations reaching approximately 1.2 × 10^6^ particles/km^2^. Red points indicate fiber-dominated sites; blue points indicate higher proportions of spheres/foam. (B) Size and aspect ratio distributions by morphological category. Fibers exhibited median lengths of 2.5–3.0 mm and widths < 0.2 mm, yielding extreme aspect ratios routinely > 50:1 (often >100:1). Fragments showed intermediate dimensions with lower aspect ratios. Differences among categories were highly significant (Kruskal–Wallis test, p < 0.0001).

Time analysis revealed a significant shift in morphological composition between 2015 and 2018. The relative abundance of fibers increased from 55% to 78% (linear regression slope = 5.8% per year, *p* = 0.012), accompanied by a corresponding decrease in fragments, while the other categories remained consistently below 6% (Fig. 1B). Spatial analysis identified significant clustering of microplastics in the Central Bay, with mean concentrations reaching approximately 1.2 × 10^6^ particles/km^2^ (Moran's index = 0.32, *p* < 0.001), indicating a non-random spatial distribution pattern (Fig. 2A).^16^

Dimensional-geometric analysis revealed significant differences depending on the morphology (Fig. 2B). Fibers had an average length of 2.5–3.0 mm and a width of less than 0.2 mm, resulting in extreme aspect ratios that often exceeded 50:1 and even reached 100:1. In contrast, fragments exhibited intermediate dimensions (length 0.5–2.0 mm; width 0.3–1.0 mm), corresponding to much lower aspect ratios. Spheres and foams were generally smaller (< 0.5 mm) and more isotropic in shape.

### 3.2 Implications for exposure and potential health relevance

To contextualize the observed environmental distribution, we assessed the potential implications of the dominant morphology of microplastics on experimentally reported biological responses. The prevalence of high-aspect-ratio fibers is significant, as these geometries encompass a wide range of sizes and thus increase the likelihood of interactions with biological systems through multiple exposure routes. Rescaling the particle size distribution using a power-law relationship estimated the total abundance of microplastics (1–5000 μm) at approximately 700,000 particles/km^2^, indicating a substantial environmental burden. Fibers exhibit a broader size spectrum than other morphologies, which may increase their persistence and behavior in aquatic ecosystems. Furthermore, their morphology may facilitate interactions with biological surfaces and cellular uptake mechanisms, as suggested by previous experimental studies. Microplastics can also act as co-carriers of pollutants, including heavy metals and hydrophobic organic compounds, further influencing their biological impact. These combined characteristics highlight the importance of considering morphology, in addition to concentration, in environmental exposure assessments.

## 4 Discussion

This study comprehensively characterizes the morphology of microplastics in a large, quality-controlled environmental dataset, identifying a clear dominance of fragments and fibers, with fibers showing a significant increasing trend over time and differing geometric properties. These results add to the growing body of evidence suggesting that the physical characteristics of microplastics, their morphology, are important determinants of their environmental behavior and potential biological interactions.

### 4.1 Fiber morphology and frustrated phagocytosis

Fibers exhibited median aspect ratios routinely exceeding 50∶1. This geometry falls within the range experimentally demonstrated to cause “frustrated phagocytosis” in macrophages, including liver-resident Kupffer cells. Studies summarized in Table 1 show that fibers beyond a critical length (approximately 15–20 μm) cannot be fully engulfed, leading to sustained reactive oxygen species production, lysosomal dysfunction, and pro-inflammatory M1 polarization.^17^

**Table 1:** Key in vitro, ex vivo, and in vivo studies demonstrating shape- and size-dependent uptake and toxicity of mps in hepatic cells.

Study (Year)	Model	MP Type & Shape/Size	Key Uptake Findings	Mechanisms/Outcomes	Relevance to Manuscript
Cheng et al.^17^ (2022)	Human liver organoids (hPSC-	PS 1 μm (irregular fragments/microbeads)	Significant hepatocyte uptake (higher than spheres)	Endocytosis → mitochondrial dysfunction → ↑ ROS (H_2_O_2_)	Human-relevant model showing hepatotoxicity and lipid
Wang et al.^18^ (2023)	Mice (in vivo, C57BL/6); macrophage-hepatocyte co-culture	PS 5 μm (spheres and elongated variants)	Macrophage uptake (70%–90%); METs transfer to hepatocytes	Frustrated phagocytosis → lysosomal damage → ROS/TGF-β/Smad2/3 activation → liver fibrotic injury; ↑ TNF-α, IL-6	Direct link between MPs, MET formation, and hepatic inflammation/fibrosis
Rudolph et al.^19^ (2021)	Murine Kupffer cells (ImKC), macrophages (J774A.1), hepatocytes (BNL CL.2)	PS spheres/fibers (0.2–6 μm)	High uptake (80–100%) in macrophages/Kupffer cells (size-dependent, optimal ∼1–2 μm); low uptake (< 20%) in hepatocytes	Phagocytosis (actin-dependent); indications of cellular stress and proliferation effects at high doses in ImKC; limited cytotoxicity except at > 250 μg/mL	Supports high macrophage/Kupffer cell interaction with microplastics; fibers discussed in broader frustrated phagocytosis context
Liu et al.^20^ (2024)	Primary mouse Kupffer cells (scRNA-seq); NAFLD model	PS 5–20 μm (fibers/fragments)	Fiber-associated Uptake; alterations in immune cell populations	PPAR/ROS Pathway activation; M1 polarization; changes in carcinogenesis-related genes with fragments	Fiber- and Fragment-specific effects on Kupffer cells and liver immune microenvironment
Koner et al.^21^ (2025)	Human THP-1 or murine RAW 264.7 macrophages	PS spheres/fibers (0.1–5 μm; focus on < 0.5 μm in some variants)	Dose- and size-dependent uptake (maximal at 1–2 μm; higher vs. spheres in some comparisons)	Oxidative stress, reduced viability, impaired immune function	Shape/size influence on macrophage impairment
Winkler et al.^22^ (2022)	Human airway organoids	Polyester/nylon fibers (10–50 μm range in related studies)	Fibers enveloped by cells; polarized growth along fibers	Reduced club cell marker (SCGB1A1); no strong inflammation/ROS in this model	Model for long-fiber interactions (note: airway-focused; supports general elongated particle effects)

Abbreviations: IL‑6, interleukin‑6; ImKC, immortalized Kupffer cells; NAFLD, non‑alcoholic fatty liver disease; PPAR, peroxisome proliferator‑activated receptor; RAW 264.7 (RAW), murine macrophage cell line; ROS, reactive oxygen species; PS, polystyrene; TGF‑β, transforming growth factor‑beta; THP‑1, human acute monocytic leukemia cell line; TNF‑α, tumor necrosis factor‑alpha.

### 4.2 Mechanistic plausibility linking observed morphologies to hepatic toxicity

Primary toxicological studies provide quantitative evidence supporting mechanistic pathways linking microplastic exposure to hepatic injury, including oxidative stress, mitochondrial dysfunction, lipid metabolic disturbance, macrophage extracellular trap formation, and fibrotic signaling.^17^^,^^18^^,^^20^ In human liver organoids derived from pluripotent stem cells, exposure to 1 μm polystyrene fragments at an environmentally relevant concentration (0.25 μg/mL ≈ 102 particles/mL) induced approximately 3-fold increases in H_2_O_2_ production, mitochondrial dysfunction, and lipid steatosis.^17^ In murine models, 5 μm elongated polystyrene particles triggered macrophage extracellular trap (MET) formation and 3- to 4-fold elevations in pro-inflammatory cytokines tumor necrosis factor-alpha (TNF-α) and interleukin-6 (IL-6) via lysosomal damage.^18^ Size-dependent effects were also evident: 5 μm particles caused more severe hepatocyte oxidative stress, elevated AST/ALT, and greater depletion of SIRT3 and SOD2 than 0.5 μm particles.^23^ These findings align closely with the dominance of high-aspect-ratio fibers (∼49.8%, aspect ratios routinely > 50:1) and irregular fragments (∼40.3%) in the San Francisco Bay dataset.

Recent systematic reviews and meta-analyses further strengthen biological plausibility. A meta-analysis of 118 vertebrate studies confirmed strong effects of microplastics on liver morphology, oxidative stress (↑ ROS/MDA; ↓ SOD/GSH), intracellular toxicity, and disrupted lipid metabolism, with oxidative stress and intracellular toxicity as the strongest signals.^17^ Another meta-analysis in fish showed highly significant increases in AST, ALT, ALP, LDH, and MDA (all *p* < 0.001) and depletion of antioxidant defenses.^24^ These pooled effects are consistent with the morphologies quantified here.

An important observation is the high prevalence of fibers with extreme aspect ratios, often exceeding 50:1. This geometric profile closely matches the range that experimental studies have shown to influence cellular uptake and clearance mechanisms. Specifically, elongated particles are associated with deficient phagocytosis in macrophages (“inhibited phagocytosis”), leading to the production of reactive oxygen species and persistent inflammatory signals. These processes are known to contribute to chronic inflammation and oxidative stress, which are key features of many liver diseases.^25^, ^26^, ^27^ Table 2 summarizes the proposed mechanisms by which MPs may influence liver disease progression and transplant outcomes. It is important to emphasize that the present study does not establish a direct causal relationship between exposure to environmental microplastics and liver disease. Rather, it demonstrates an association between the dominant environmental morphology and experimentally identified shape-dependent biological responses, providing a plausible biological framework for future studies. This distinction is important because the current analysis is based on environmental incident data rather than direct toxicological or clinical evidence.^28^

**Table 2:** Proposed pathways by which microplastics may exacerbate liver disease and complicate liver transplantation.

Pathway	Mechanism	Impact on Liver Disease	Key Supporting Evidence
Oxidative stress	ROS production; mitochondrial dysfunction; depletion of antioxidants (e.g., SOD, GSH, SIRT3)	Promotes hepatocyte injury, steatosis, and fibrosis (MAFLD/MASLD-like changes)	Cheng et al. (2022)^17^– ↑ ROS and lipid disruption in human liver organoids; meta-analyses Zhang et al. (2024)^29^ and Sun et al. (2024)^24^ showing consistent oxidative stress signals
Inflammatory response	Cytokine release (↑ TNF-α, IL-6); frustrated phagocytosis in Kupffer cells; macrophage extracellular traps (METs); M1 polarization	Exacerbates chronic inflammation, NAFLD/MAFLD progression, and fibrotic injury	Wang et al. (2023)^18^ – METs and ROS/TGF-β/Smad axis in liver fibrosis; Liu et al. (2024)^20^ – Kupffer cell changes
Gut-liver axis disruption	Altered microbiota; increased LPS translocation; synergistic pollutant carriage	Amplifies hepatic inflammation and metabolic dysfunction	Zheng et al. (2021)^13^ and Zhang et al. (2024)^30^ support a gut–liver axis mechanism, showing that microplastic exposure is associated with intestinal inflammation, gut microbiota/metabolite disruption, and secondary hepatic injury.
Impaired immune regulation	Sustained macrophage activation; potential Treg dysfunction	May worsen autoimmune or chronic liver conditions; increased susceptibility to injury	Supported by experimental evidence demonstrating MET formation, Kupffer cell M1 polarization, and persistent macrophage activation following microplastic exposure (Wang et al., 2023);^18^ Liu et al., 2024).^20^
Interference with hepatic metabolism	Modulation of CYP450 enzymes; disrupted lipid/glucose pathways	Alters detoxification and lipid homeostasis; potential interaction with therapeutics	Cheng et al. (2022);^17^ animal models showing enzyme and metabolic gene changes

**Abbreviations:** CYP450, cytochrome p450; GSH, glutathione; IL-6, interleukin-6; LPS, lipopolysaccharide; MAFLD, metabolic associated fatty liver disease; MASLD, metabolic dysfunction-associated steatotic liver disease; MET, metformin; MPs, microplastics; NAFLD, non-alcoholic fatty liver disease; SIRT3, sirtuin 3; SOD, superoxide dismutase; TNF-α, tumor necrosis factor-alpha.

Fragments, the second most common morphological class, exhibit characteristics that could be biologically relevant. Their irregular geometry and intermediate size range have been associated in previous studies with increased cellular stress responses, including mitochondrial dysfunction and lysosomal instability.^18^, ^19^, ^20^, ^21^ Compared to spherical particles, irregular shapes may lead to greater mechanical interactions with cellular structures, which could amplify adverse side effects. The observed increase in fiber dominance over time may reflect broader trends in synthetic textile production and release into the environment, suggesting that human exposure to these morphologies may be increasing over time.^21^^,^^22^ Due to the persistence and mobility of fibers, their accumulation in environmental compartments warrants further attention.

Despite these considerations, some limitations should be recognized. The use of a 333 μm mesh in the original dataset likely underestimated smaller microplastics and nano-plastics, which may have different biological properties. Furthermore, the analysis was limited to a single geographic region, which may limit its generalizability, although similar morphological patterns have been reported worldwide. The study also did not stratify the results by polymer type, which may have an independent impact on toxicity. More importantly, the proposed health implications are inferential and based on integration with existing experimental literature, rather than direct evidence from human studies. Future research should aim to fill this gap by integrating environmental exposure data with toxicological and clinical studies. Studies focusing on dose-response relationships, tissue-specific accumulation, and long-term effects of environmentally relevant microplastic mixtures will be key. The accuracy of health risk assessment could be significantly improved if morphology were considered as a critical parameter in such studies.

## 5 Conclusion

This study shows that microplastics in water are mainly caused by fragments and fibers with a high aspect ratio and that these fibers grow significantly over time. The geometric characteristics of these particles are consistent with experimentally reported mechanisms, including impaired phagocytic processes and oxidative stress. Although a direct causal relationship with liver disease cannot be established, the observed association between environmental morphology and biological response mechanisms highlights the importance of considering particle shape in environmental exposure assessment models. Future studies that integrate environmental, toxicological, and clinical data will be essential to clarify the health consequences of exposure to microplastics.

## Informed consent

Not Applicable.

## Ethics statement

Not Applicable.

## Organ donation

The authors have nothing to report.

## Animal treatment

The authors have nothing to report.

## Data availability statement

The data underlying this article are publicly available from the California Water Boards Microplastic Data repository via https://Data.gov: https://catalog.data.gov/dataset/microplastic-and-microparticle-data-from-surface-water-san-francisco-bay-and-adjacent-sanc. The data underlying this article will be shared on reasonable request from the corresponding author.

## Declaration of generative AI and AI-assisted technologies in the writing process

During the preparation of this work, the authors used DeepSeek-R1 to assist with English language polishing. After utilizing this tool, the authors thoroughly reviewed and edited the content as needed. The authors take full responsibility for all aspects of the publication's content.

## Funding

This study was supported by 10.13039/501100001809National Natural Science Foundation of China (82270795) and Hainan Province 10.13039/100018696Health Science and Technology Innovation Joint Key Project (WSJK2025ZD214).

## CRediT authorship contribution statement

**Muhammad Adil Malik:** Conceptualization, Data curation, Formal analysis, Investigation, Methodology, Project administration, Resources, Software, Validation, Visualization, Writing – original draft, Writing – review & editing. **Quan Zhuang:** Funding acquisition, Supervision.

## Declaration of competing interest

The authors declare no conflict of interest. All authors declare that the funders did not participate in the research design, data collection, analysis, interpretation, or the writing of the paper.
